# Supplementary material for: Revisiting Genetic Epidemiology with a Refined Targeted Gene Panel for Hereditary Hearing Impairment in the Taiwanese Population
Source: Genes (Basel). 2023 Apr 7;14(4):880. doi: 10.3390/genes14040880 (PMC10137978; doi:10.3390/genes14040880)
Supplement: Supplementary file 1 [file genes-14-00880-s001.zip › genes-2290782-supplementary.pdf]

Table S1. NCBI reference sequences of the 30-gene panel.

	NCBI Reference Sequence
AIFM1	NM_004208 / NP_004199
DIAPH3	NM_001042517 / NP_001035982
EDN3	NM_000114 / NP_996917
EDNRB	NM_001201397 / NP_001188326
EYA1	NM_000503 / NP_000494
FOXI1	NM_012188 / NP_036320
GJA1	NM_000165 / NP_000156
GJB1	NM_001097642 / NP_001091111
GJB2	NM_004004 / NP_003995
GJB3	NM_024009 / NP_076872
GJB4	NM_153212 / NP_694944
GJB6	NM_006783 / NP_006774
KCNJ10	NM_002241 / NP_002232
KCNQ4	NM_004700 / NP_004691
MITF	NM_198159 / NP_001171896

Continued on the next page

Table S1 (con’t). NCBI reference sequences of the 30-gene panel.

	NCBI Reference Sequence
MYO15A	NM_016239 / NP_057323
OTOF	NM_004802 / NP_004793
PAX3	NM_181459 / NP_852124
PCDH9	NM_203487 / NP_982354
POU3F4	NM_000307 / NP_000298
POU4F3	NM_002700 / NP_002691
PJVK	NM_001042702 / NP_001036167
SIX1	NM_005982 / NP_005973
SIX5	NM_175875 / NP_787071
SLC26A4	NM_000441 / NP_000432
SNAI2	NM_003068 / NP_003059
SOX10	NM_006941 / NP_008872
STRC	NM_153700 / NP_714544
MT-RNR1 / MT-TL1	NC_012920

Table S2. Causative variants of the relevant genes (GJB2).

Gene	Variants	Number of Patients
	c.109G>A (p.V37I)/ c.109G>A (p.V37I)	89
	c.109G>A (p.V37I)/c.235delC (p.L79Cfs *3)	6
	c.235delC (p.L79Cfs *3)/ c.235delC (p.L79Cfs *3)	5
	c.109G>A (p.V37I)/c.101T>C (p.M34T)	1
	c.109G>A (p.V37I)/c.283G>A (p.V95M)	1
GJB2	c.109G>A (p.V37I)/c.571T>C (p.F191L)	1
	c.109G>A (p.V37I)/c.609C>G (p.I203M)	1
	c.109G>A (p.V37I)/c.494G>A (p.R165Q)	1
	c.235delC (p.L79Cfs *3)/c.35dup (p.V13fs)	1
	c.223C>T (p.R75W)	1
	c.508_511dupAACG (p.A171Qfs *40)	1

Table S3. Causative variants of the relevant genes (SLC26A4 and OTOF).

Gene	Variants	Number of Patients
SLC26A4	c.919-2A>G/ c.919-2A>G	15
	c.919-2A>G/c.1975G>C (p.V659L)	3
	c.919-2A>G/c.2168A>G (p.H723R)	2
	c.919-2A>G/c.1160C>T (p.A387V)	1
	c.919-2A>G/c.1174A>T (p.N392Y)	1
	c.919-2A>G/c.1225C>T (p.R409C)	1
	c.919-2A>G/c.1229C>T (p.T410M)	1
	c.919-2A>G/c.1271G>A(p.G424D)	1
	c.919-2A>G/c.1716T>A (p.F572L)	1
	c.919-2A>G/c.1786C>T (p.Q596Ter)	1
	c.919-2A>G/c.751_752delCTinsTC (p.Leu251Ser)	1
	c.1262A>C (p.Q421P)/c.1707+5G>A	1
OTOF	c.5098G>C (p.E1700Q)/ c.5098G>C (p.E1700Q)	3
	c.5098G>C (p.E1700Q)/c.5975A>G (p.K1992R)	4

Table S4. Causative variants of the relevant genes (MYO15A)**.**

Gene	Variants	Number of Patients
MYO15A	c.3524dup (p.S1176fs)/ c.3524dup (p.S1176fs)	2
	c.3524dup (p.S1176fs)/c. 8828dupT (p.S2945Ffs*55)	1
	c.3524dup (p.S1176fs)/c.10250_10252delCCT (p.S3417del)	1
	c.3524dup (p.S1176fs)/c.3757-32_3757-1del	1
	c.3524dup (p.S1176fs)/c.6863C>T (p.S2288L)	1
	c.3524dup (p.S1176fs)/c.6956+1G>A	1
	c.3524dup (p.S1176fs)/c.7708_7709insCA (p.Q2571Hfs*35)	1
	c.3742C>T (p.R1248W)/c.4304A>C (p.Y1435S)	1
	c.3926A>T (p.Q1309L)/c.10250_10252delCCT (p.S3417del)	1
	c.4642G>A (p.A1298T)/c.3958G>A (p.G1320S)	1
	c.7711_7712dupCA (p.Gln2571HisfsTer35)/c. 8340+5G>A	1
	c.10250_10252delCCT (p.S3417del)/c.10258_10260delTTC (p.F3420del)	1
